# Humoral response to SARS-CoV-2 mRNA vaccine on in ABO blood type incompatible kidney transplant recipients treated with low-dose rituximab

**DOI:** 10.1038/s41598-023-42406-5

**Published:** 2023-09-12

**Authors:** Tomoko Hamaya, Shingo Hatakeyama, Tohru Yoneyama, Yuki Tobisawa, Hirotake Kodama, Takeshi Fujita, Reiichi Murakami, Kazuyuki Mori, Teppei Okamoto, Hayato Yamamoto, Takahiro Yoneyama, Yasuhiro Hashimoto, Hisao Saitoh, Shunji Narumi, Hirofumi Tomita, Chikara Ohyama

**Affiliations:** 1 Department of Urology, Hirosaki University School of Medicine, 5 Zaifu-cho, Hirosaki, Aomori 036-8562 Japan; 2 Department of Advanced Blood Purification Therapy, Hirosaki University School of Medicine, 5 Zaifu-cho, Hirosaki, Aomori 036-8562 Japan; 3 Department of Glycotechnology, Center for Advanced Medical Research, Hirosaki University School of Medicine, 5 Zaifu-cho, Hirosaki, Aomori 036-8562 Japan; 4 Department of Cardiology and Nephrology, Hirosaki University School of Medicine, 5 Zaifu-cho, Hirosaki, Aomori 036-8562 Japan; 5 Department of Advanced Transplant and Regenerative Medicine, Hirosaki University School of Medicine, 5 Zaifu-cho, Hirosaki, Aomori 036-8562 Japan; 6 Department of Urology, Oyokyo Kidney Research Institute, 90 Kozawayamazaki, Hirosaki, Aomori 036-8243 Japan; 7 Department of Transplant Nephrology and Surgery, Japanese Red Cross Aichi Medical Center Nagoya Daini Hospital, Nagoya, Japan

**Keywords:** Immunology, Infectious diseases, Transplant immunology, Kidney, Renal replacement therapy, Infectious diseases, Kidney diseases

## Abstract

We aimed to evaluate the humoral response after the second and third doses of SARS-CoV-2 mRNA vaccine in ABO blood type incompatible kidney transplant (KT) recipients treated with rituximab. This retrospective study conducted between June 2021 and June 2022 included 131 KT recipients and 154 nontransplant controls who had received mRNA vaccines. We compared the seropositivity (anti-SARS-CoV-2 spike IgG antibody titer ≥ 0.8 U/mL) after the second and third vaccinations. Furthermore, we evaluated the impact of pretransplant vaccination for seropositivity. Of the 131 KT recipients, 50 had received the third dose of mRNA vaccine. The antibody titer was significantly increased after the third dose of mRNA vaccine. The seropositivity rate after the third dose of mRNA vaccine increased from 36 to 70%. We observed no significant difference in seropositivity after the third dose of mRNA vaccine in ABO incompatibility, rituximab use, mycophenolate mofetil use, and age at KT. Of the nine recipients who had received the second or third dose of the mRNA vaccine prior to the KT, eight of the recipients were seropositive both before and after the KT. Our results suggest that ABO incompatibility or rituximab use was not significantly associated with seropositivity.

## Introduction

Kidney transplant (KT) recipients have a high mortality rate for severe acute respiratory syndrome coronavirus 2 (SARS-CoV-2) infection^1^. Although these patients are prioritized for vaccination, a diminished seropositivity rate after the second dose of mRNA vaccine against the receptor-binding domain of SARS-CoV-2 spike (S) protein has been observed in KT recipients (ranging from 32 to 54%), as compared to nontransplant controls (98%–100%)^2–9^, but it improved significantly following the third dose of vaccine (ranged from 29.4 to 80.6%)^10–18^. However, most studies evaluating immunoglobulin G (IgG) antibody titer after the third dose of mRNA vaccine in KT recipients were from Western countries^2–8,10–15^. As KT protocols vary across countries and regions, the humoral response to the third dose of mRNA vaccine in ABO blood type incompatible (ABO-incompatible) KT recipients with low-dose rituximab has not been validated. Furthermore, limited data are available on the impact of pretransplant vaccination on seropositivity and IgG antibody titer in KT recipients. Therefore, we aimed to measure the humoral response after the third dose of the Pfizer/BioNTech BNT162b2 or Moderna mRNA-1273 vaccine and to investigate the effect of pretransplant vaccination on seropositivity in KT recipients.

## Results

### Background of patients

**Figure 1:**
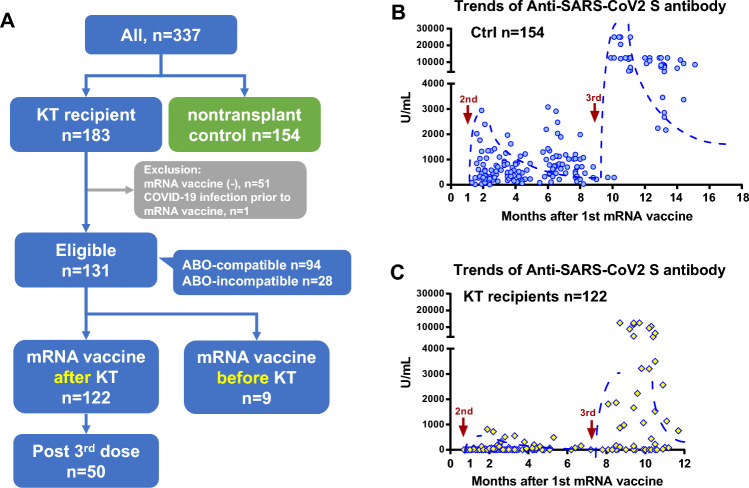
The proportion of eligible participants and trends in anti-SARS-CoV-2 IgG S antibody titers after the mRNA vaccination. (**A**) The proportion of eligible participants who had received the SARS-CoV-2 mRNA vaccines. (**B**) Trends in anti-SARS-CoV-2 IgG S antibody titers after the mRNA vaccination in the control group (n = 154). (**C**) Trends in anti-SARS-CoV-2 IgG S antibody titers after the mRNA vaccination in kidney transplant recipients who had received the mRNA vaccine before kidney transplantation (n = 122). *SARS-CoV-2* severe acute respiratory syndrome coronavirus 2, *S* spike, *IGG* immunoglobulin G.

Figure 1A summarizes the characteristics of the patients incorporated in this study. This study included 154 nontransplant controls, 122 K recipients who had received the mRNA vaccine after KT, and 9 recipients who had received the mRNA vaccine before KT. Of the 122 recipients who had received the mRNA vaccine after KT, 50 had received the third dose of mRNA vaccine after KT. 68 The median time between the second and third vaccination was 7 months. The other 82 recipients were not received the third dose of mRNA vaccine at the end of June 2022. Recipients with a previous SARS-Cov-2 infection were not included. The background characteristics of the study cohort are summarized in Table 1. The number of ABO-incompatible KT recipients and the number of recipients treated with low-dose rituximab were 28 and 50, respectively.

**Table 1:** Background of participants.

	Nontransplant Ctrl	KT recipient	
		mRNA vaccine after KT	mRNA vaccine before KT
Number, n	154	122	9
Age, years (IQR)	64 (36, 76)	43 (33, 58)	52 (28, 59)
60 years old or older, n	78 (50.6%)	54 (44.3%)	2 (22.2%)
Male, n	100 (65%)	72 (59%)	7 (78%)
ABO blood type incompatible KT, n (%)		28 (23%)	3 (33%)
Rituximab use, n (%)		50 (41%)	5 (56%)
Post-3rd dose of vaccination, n	46 (30%)	50 (41%)	1 (11%)
Median months from 1st-vaccine to KT (IQR)			4.4 (2.2–5.9)

*KT* kidney transplant, *IQR*:interquartile range.

The median dosages of maintenance immunosuppression in tacrolimus, mycophenolate mofetil (MMF), and prednisolone were 3.0 mg (IQR 2.0–4.5 mg), 1000 mg (IQR 750–1000 mg), 5 mg as (IQR 5.0–5.0 mg). Everolimus was administrated in 10 patients with the median dose of 1.5 mg (IQR 1.0–1.5 mg) The median trough level of tacrolimus was 3.9 mg (IQR 3.1–4.9 ng/mL).

Of the 50 recipients who had received the third dose of mRNA vaccine after KT, rituximab was administrated in 21 (42%) KT recipients, including 7 (14%) ABO-incompatible KT recipients. All recipients received combined immunosuppressive therapy with a median of three agents. The median period after KT was 5.8 (IQR 2.9, 10.3) years. No recipient had biopsy-proven rejection or viral events during the current study period.

### Trends in anti-SARS-CoV-2 S IgG antibody

The third dose of mRNA vaccine caused a marked increase in antibody titers in both nontransplant controls (Fig. 1B) and KT recipients (Fig. 1C). The median IgG antibody titers in the nontransplant controls before and after the third dose of mRNA vaccine were 593 and 9110 U/mL, respectively (*P* < 0.001). All nontransplant controls were seropositive (100%).

### Antibody titer and seropositivity

**Figure 2:**
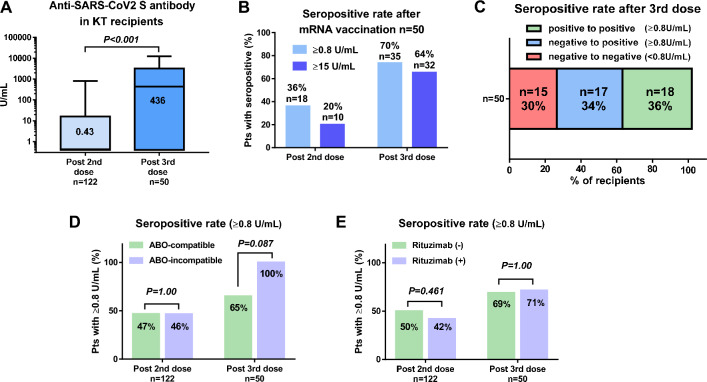
Comparison of anti-SARS-CoV-2 IgG S antibody titers and seropositivity rates after the second and third doses of the mRNA vaccine in kidney transplant recipients. (**A**) A comparison of antibody titers after the second and third doses of the mRNA vaccine in kidney transplant (KT) recipients. (**B**) A comparison of the seropositivity rate after the second and third doses of the mRNA vaccine in KT recipients (n = 50). Seropositivity was defined as anti-SARS-CoV-2 S IgG antibody titers of ≥ 0.80 or ≥ 15 U/mL. (**C**) Changes in seropositivity from the second to third dose of the mRNA vaccine in KT recipients (n = 50). (**D**) A comparison of the seropositivity rate after the second (n = 122) and third (n = 50) doses of the mRNA vaccine between the ABO-compatible and ABO-incompatible KT recipients. (**E**) A comparison of the seropositivity rate after the second (n = 122) and third (n = 50) doses of the mRNA vaccine between the KT recipients with and without rituximab. *SARS-CoV-2* severe acute respiratory syndrome coronavirus 2, *S* spike, *IGG* immunoglobulin G.

In KT recipients, the anti-SARS-CoV-2 S IgG antibody titer was significantly increased after the third dose of mRNA vaccine, as compared to that during the post-second dose (436 vs. 0.43 U/mL, *P* < 0.001; Fig. 2A). The rates of anti-SARS-CoV-2 S IgG antibody titer level ≥ 0.8 and ≥ 15 U/mL were 70% (n = 35/50) and 64% (n = 32/50), respectively in the KT recipients. The seropositivity rates were significantly higher after the third dose than after the second dose (*P* = 0.001 and *P* < 0.001, respectively; Fig. 2B). Of the 50 recipients, 18 (36%) and 32 (64%) were seropositive and seronegative, respectively, before the third dose of the mRNA vaccine. However, 17 recipients (34%) who were seronegative after the second dose became seropositive after the third dose of the mRNA vaccine (Fig. 2C). There were no significant differences in the background characteristics between the KT recipients with seronegativity (n = 15) and seropositivity (n = 35) after the third of the mRNA vaccine, except for any responses in the anti-SARS-CoV-2 S IgG antibody titer to the second dose of the mRNA vaccine, which was significantly associated with seropositivity after the third dose of the mRNA vaccine (Table 2).

**Table 2:** Background of KT recipients who administrated the third dose of mRNA vaccine.

	Negative (< 0.8 U/mL)	Positive (> = 0.8 U/mL)	*P* value
Number, n	15	35	
Age at KT, years (IQR)	55 (40, 58)	40 (32, 55)	0.133
Age at the 1st-mRNA vaccine, years (IQR)	60 (40, 65)	49 (43, 62)	0.093
Male, n (%)	9 (60%)	22 (63%)	1.000
Median KT vintage, years	5.4 (2.5, 10.9)	6.4 (3.4, 9.8)	0.675
KT from deceased donor, n (%)	2 (13%)	7 (20%)	0.705
Interval from the 1st to 3rd dose, months (IQR)	8.0 (7.5, 8.5)	8.1 (7.8, 8.5)	0.549
Interval from the 2nd to 3rd dose, months (IQR)	7.3 (6.8, 7.8)	7.5 (7.1, 7.8)	0.618
Primary kidney disease, n (%)			
Glomerular	5 (33%)	19 (54%)	0.224
Diabetes	2 (13%)	4 (11.4%)	1.000
Others	8 (53%)	12 (35%)	0.228
ABO blood type incompatible KT, n (%)	0 (0%)	7 (20%)	0.087
Immunosuppressant regimen, n (%)			
Tacrolimus	14 (93%)	30 (86%)	0.654
Cyclosporine	1 (6.7%)	2 (5.7%)	1.000
Mycophenolate mofetil	14 (93%)	30 (86%)	0.654
Azathioprine	1 (6.7%)	2 (5.7%)	1.000
Everolimus	2 (13%)	5 (14%)	1.000
Steroids	14 (93%)	34 (97%)	0.514
Rituximab	6 (40%)	15 (43%)	1.000
Any history of rejection events, n (%)	1 (6.7%)	0 (0%)	0.300
History of viral infection events except for COVID-19, n (%)	2 (13%)	4 (11.4%)	1.000
eGFR at vaccination, mL/min/1.73m^2^	38 (33, 44)	47 (34, 54)	0.105
Any response to the 2nd-mRNA vaccine, n (%)	0 (0%)	18 (51.4%)	< 0.001

*KT* kidney transplant, *eGFR* estimated glomerular filtration rate, *IQR* interquartile rang.

### Seropositivity in ABO-incompatible KT recipients

The seropositivity rate after the second dose of mRNA vaccine was not significantly different between the ABO-compatible (n = 44/94, 46.8%) and ABO-incompatible (n = 13/28, 46.4%) KT recipients (*P* = 1.00). The seropositivity rate after the third dose of mRNA vaccine was not significantly different between the ABO-compatible (n = 28/43, 65.1%) and ABO-incompatible (n = 7/7, 100%) KT recipients (*P* = 0.087) (Fig. 2D).

### Seropositivity in KT recipients with or without rituximab

The seropositivity rate after the second dose of mRNA vaccine was not significantly different between the KT recipients without rituximab (n = 36/72, 50.0%) and with rituximab (n = 21/50, 42.0%) (*P* = 0.461). The seropositivity rate after the third dose of mRNA vaccine was not significantly different between the KT recipients without rituximab (n = 20/29, 69.0%) and with rituximab (n = 15/21, 71.4%) (*P* = 1.00) (Fig. 2E).

### Impact of vaccinations prior to transplantation

**Figure 3:**
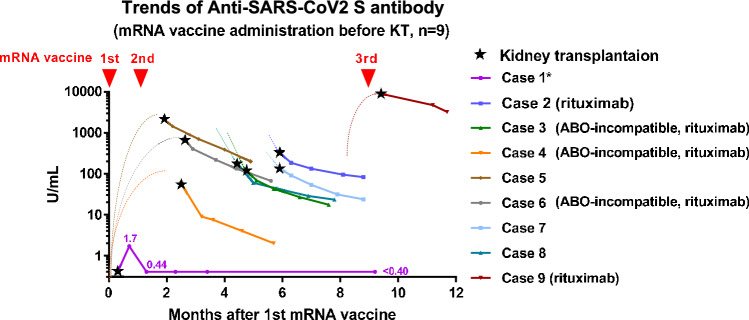
Trends in anti-SARS-CoV-2 IgG S antibody titers in each kidney transplant recipient who had received the mRNA vaccine before kidney transplantation. *, case 1: a single dose of the MRNA vaccine before KT, followed by second and third doses after KT. *SARS-CoV-2* severe acute respiratory syndrome coronavirus 2, *S* spike, *IGG* immunoglobulin G, *KT* kidney transplantation.

The trends in anti-SARS-CoV-2 S IgG antibody titer in the recipients who had received the mRNA vaccine before KT (n = 9) showed that eight recipients who had received the second or third dose of the mRNA vaccine were seropositive, whereas one recipient who had received only a single dose before KT showed seronegativity after KT even if the recipient was vaccinated later (Fig. 3). No serious adverse event was observed in patients receiving a third vaccination.

### COVID-19 infection event

**Figure 4:**
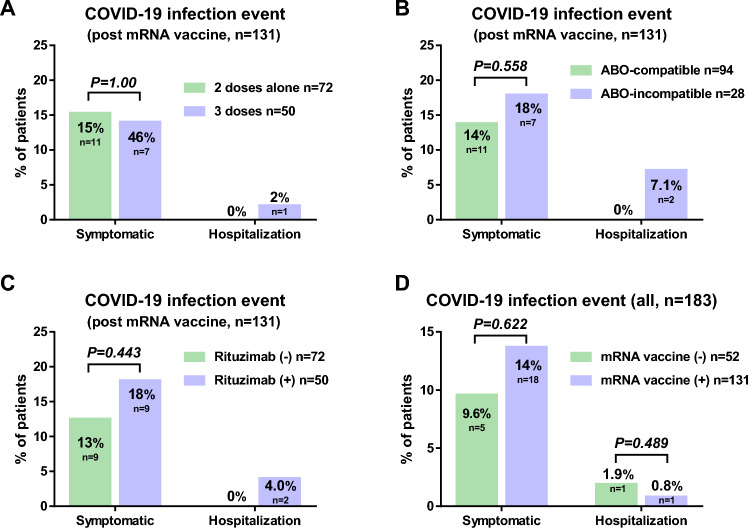
Comparison of COVID-19 infection event in kidney transplant recipients. (**A**) A comparison of COVID-19 infection event between the kidney transplant (KT) recipients who received two doses alone and those who received three doses of mRNA vaccine. (**B**) A comparison of COVID-19 infection event between the ABO-compatible and ABO-incompatible KT recipients. (**C**) A comparison of COVID-19 infection event between the KT recipients with rituximab and those without rituximab. (**D**) A comparison of COVID-19 infection event between the KT recipients with mRNA vaccine and those without mRNA vaccine.

We evaluated COVID-19 infection events between June 21, 2021 and December 31, 2022. We observed no significant difference in the rate of symptomatic COVID-19 infection events between patients on 3 doses (n = 11/72, 15.3%) and those on 2 doses alone (n = 7/50, 14%) (*P* = 1.00). The rate of hospitalization was 0% in patients on 3 doses and 2 doses alone (n = 1, 2%) (Fig. 4A). There were no deaths related to COVID-19 during this period. The rate of symptomatic COVID-19 infection events was not significantly different between patients with ABO-compatible KT (n = 13/94, 13.8%) and those with ABO-incompatible KT (n = 5/28, 17.9%) (*P* = 0.558) (Fig. 4B) and between patients with rituximab (n = 9/50, 18%) and those without rituximab (n = 9/72, 12.5%) (*P* = 0.443) (Fig. 4C). Among all KT recipients (n = 183), the rates of symptomatic COVID-19 infection with and without mRNA vaccines were 13.7% (n = 18/131) and 9.6% (n = 5/52), respectively (*P* = 0.622). The rates of hospitalization with and without mRNA vaccine were 0.8% (n = 1/131) and 1.9% (n = 1/52), respectively (*P* = 0.489) (Fig. 4D).

## Discussion

In the present study, when evaluating the humoral response after the third dose of the mRNA vaccine in KT recipients in Japan, 70% and 65% of the KT recipients had anti-SARS-CoV-2 IgG antibody titers of ≥ 0.8 and ≥ 15 U/mL, respectively. This finding is comparable to those of previous studies reporting the humoral response after the third dose in KT recipients^10–15^. However, the rate of anti-SARS-CoV-2 IgG seropositivity varies across the studies (ranged from 29.4 to 80.6%) owing to the differences in study population and measurement methods^19^. Accordingly, the U.S. Food and Drug Administration states that “antibody testing is not currently recommended” because the clinical implication of SARS-CoV-2 antibody tests to assess immunity after COVID-19 vaccination remains unknown^20^. Furthermore, the the anti-SARS-CoV-2 S IgG titer cut-off values of ≥ 0.8 and ≥ 15 U/mL may not be entirely protective levels post-vaccination in the context of Delta and Omicron variants^21–24^. These findings highlight the need for careful interpretation of the results of anti-SARS-CoV-2 S IgG titers in KT recipients. Further studies are needed to determine the optimal cutoff values and efficacy of SARS-CoV-2 mRNA vaccines in KT recipients. Moreover, the efficacy of SARS-CoV-2 vaccines cannot be measured by IgG antibody titers alone.

We identified that 32 out of 50 recipients were seronegative before the third dose of the mRNA vaccine. Of the 32 recipients with seronegativity, 17 (53%) obtained seroconversion after the third dose of vaccine. This finding was similar to those of previous studies evaluating seropositivity after the third dose of vaccine (29.4–52.5%)^12,14,15,25^. Given that 30% of KT recipients were seronegative even after the third dose of vaccine, it may lead to the necessity for the fourth or fifth vaccination. A previous study evaluated the humoral response to three, four, and five doses of vaccine in KT recipients who were seronegative before each dose. The cumulative humoral response increased from 19.1%, 42.0%, 74.2%, and 88.7% after the second, third, fourth, and fifth vaccinations, respectively. These results may suggest the potential benefit of repeat vaccination in immunosuppressive individuals to boost their humoral response. However, the efficacy of the current mRNA vaccine against wild-type SARS-CoV-2 S protein is questionable for the Omicron variants. A recent study evaluated neutralizing antibodies against SARS-CoV-2 variants, including the Delta and Omicron variants, following the third dose of mRNA vaccine^13^. They demonstrated a greatly diminished seropositivity rate to the Delta (59%) and Omicron (12%) variants, while 61% had enough antibodies against the wild-type variant^13^. Further study is necessary to address the benefit of repeat vaccination in KT recipients who have an impaired humoral response.

The factors for impaired humoral response to the third dose of the mRNA vaccine are not well-known. A previous study evaluating repeat vaccination for KT recipients (n = 574 for the third dose) suggested that any previous slight response in anti-SARS-CoV-2 S IgG antibody titer, younger age, higher body mass index, older age at transplantation, higher estimated glomerular filtration rate (eGFR), and higher hemoglobin levels were associated with an improved humoral response after three doses of SARS-CoV-2 vaccine^15^. Although we did not find any difference in the background characteristics between seronegative (n = 15) and seropositive (n = 35) KT recipients after the third dose of the mRNA vaccine, any responses to the second dose were significantly associated with seropositivity after the third dose of mRNA vaccine. Although age, immunosuppressive therapy [rituximab or MMF use], ABO blood-type compatibility, and KT vintage were not found to be associated with an impaired humoral response after the third dose of SARS-CoV-2 mRNA vaccination; this finding might be attributed to small sample size analyzed in this study. Moreover, the long-time from transplantation to vaccination might explain the lack of influence of rituximab.

Regarding the evaluation of symptomatic COVID-19 infection events, patients on the two-dose group have a longer follow up than patients with the third dose. That the risk of infection is probably higher irrespective of vaccination. Although there was no significant difference in the rate of symptomatic COVID-19 infection events between patients of two and three doses of vaccine, these results should be interpreted with caution.

Vaccination prior to transplantation plays a key role^26^. Recipients who received at least two doses of the mRNA vaccine prior to KT maintained high antibody titers after KT, regardless of immunosuppressive regimens. It should be noted that all recipients who had received the second dose of the mRNA vaccine (n = 8) were seropositive, while one recipient who had only received a single dose before KT remained seronegative. A third dose could not promote the humoral response in this recipient (case 1 in Fig. 3). The observations support the importance of two doses of vaccinations prior to KT.

The present study included several limitations. The limited sample size and retrospective study design are robust limitations that cannot be ignored. The influence of antibody titer decline over time may affect the result because the measurement periods of antibodies are not aligned. Currently, no useful standard cut-off value is available since the multiple variants of SARS-CoV-2 has been evolving. The measurement of antibody titers is not the definitive method for assessing immunologic response and protective level post-vaccination. Furthermore, a lack of cell-mediated immune measurement are limitations in this study. We could not exclude asymptomatic SARS-CoV-2 infection because antibody to the nucleocapsid (N)-protein was not measured. Despite these limitations, this study revealed the seroprevalence of SARS-CoV-2 S IgG antibodies after the third dose of the mRNA vaccine in Japanese KT recipients including ABO-incompatible recipients and recipients treated with low-dose rituximab. Two or more vaccinations prior to transplantation may help maintain adequate seropositivity after KT transplantation.

In conclusion, we confirmed that the rate of anti-SARS-CoV-2 IgG seroconversion was 70% in KT recipients after the third dose of the mRNA vaccine. ABO incompatibility or rituximab use was not significantly associated with seropositivity and COVID-19 infection events. Further investigation is warranted to elucidate the efficacy of the third or more vaccination in patients receiving immunosuppressive therapy.

## Methods

The Ethics Committee of Hirosaki University approved this retrospective study (2021-089). All participants had provided written informed consent for other biomarker studies previously. This clinical research is consistent with the Principles of the Declaration of Istanbul and Helsinki.

### Participants

The current study conducted between June 21, 2021 and June 30, 2022 included 131 KT recipients and 154 nontransplant controls who had received the second or third dose of the mRNA vaccine (the Pfizer/BioNTech BNT162b2 or Moderna mRNA-1273 vaccine). Cumulative numbers of alive KT recipients in the transplant program were 183 at the end of June 2022. Of 183, we excluded 1 recipient who had SARS-CoV-2 infection prior to SARS-CoV-2 mRNA vaccine. Of 182, We included 131 KT recipients who had received mRNA vaccine (72.0%). The control group included members of the medical staff, medical students, and cured patients with localized cancers who were not actively receiving any treatment. Those with a previous SARS-CoV-2 infection were not included. The clinical parameters of age, sex, primary kidney disease, KT vintage (years), dialysis vintage (years), ABO blood-type compatibility, immunosuppressant agents, history of rejection events, history of viral events, and renal function (eGFR) were obtained from the medical records.

### Immunosuppression

Flow cytometry and Luminex-based single-antigen bead assay were used to select the immunosuppressive therapy protocol^27^. Basic immunosuppression included the use of calcineurin inhibitors (CNIs), MMF, steroids, and anti-CD25 monoclonal antibody basiliximab. Low-dose rituximab (100 mg/m^2^ or 100 mg/body) was administered in recipients who had donor-specific human leukocyte antigen antibodies. ABO blood-type incompatible KT recipients received basic immunosuppressive agents, rituximab, low-dose rituximab (100 mg/m^2^ or 100 mg/body), and therapeutic apheresis. Those with a viral infection or malignancies were switched from CNIs or MMF to everolimus. Page 8 line 225.

The mRNA vaccine was administered as a mass or individual vaccination; the Pfizer/BioNTech BNT162b2 was used for the first and second doses (0.03 mg/0.3 mL, deltoid muscle injection), and Moderna mRNA-1273 was also used from the third dose (0.05 mg/0.25 mL, deltoid muscle injection), but mostly Pfizer was used more frequently. The first dose was started on February 17, 2021; the second dose was given 3 weeks apart, starting on March 10, 2021; the third dose began on December 1, 2021. The third vaccination was recommended 6–8 months after the second dose by government policy due to vaccine shortages and preparation.

### Measurement of anti-SARS-CoV-2 IgG antibody titers

We underwent 1 to 5 blood tests per one patient after each vaccination time point. We measured the IgG antibodies against the SARS-CoV-2 S protein receptor-binding domain. The blood samples collected for regular evaluation were stored and used for analysis. The anti-SARS-CoV-2 S IgG antibody titer was quantitatively detected with a double-antigen sandwich-based electrochemiluminescence immunoassay (ECLIA), using the Elecsys® Anti-SARS-CoV-2 S RUO (Roche Diagnostics, Mélan, France). According to the manufacturer’s data, the values above 0.80 U/mL are considered to indicate seropositivity and the values above 15 U/mL had a likelihood of 100% to confer in vitro neutralization to SARS-CoV-2. We defined seropositivity as an antibody of ≥ 0.8 U/mL.

### Outcomes

We evaluated the trends in anti-SARS-CoV-2 S IgG antibody titer in nontransplant controls and KT recipients. The seropositivity rates after the second and third doses of the mRNA vaccine in KT recipients were compared. Furthermore, we evaluated the impact of pretransplant vaccination on seropositivity. COVID-19 infection events were evaluated between June 21, 2021 and December 31, 2022.

### Statistical analysis

Qualitative and quantitative variables were described as numbers with percentages and medians with IQRs, respectively. The Fisher’s exact, Mann–Whitney *U*, and Student’s t tests were used for the statistical comparison. All statistical analyses were performed using BellCurve for Excel 3.10 (Social Survey Research Information, Tokyo, Japan) and GraphPad Prism 7.00 (GraphPad Software, San Diego, CA, USA).

## Data Availability

Our data can be shared on reasonable request: E-mail: shingoh@hirosaki-u.ac.jp.
